# First infection by all four non-severe acute respiratory syndrome human coronaviruses takes place during childhood

**DOI:** 10.1186/1471-2334-13-433

**Published:** 2013-09-16

**Authors:** Weimin Zhou, Wen Wang, Huijuan Wang, Roujian Lu, Wenjie Tan

**Affiliations:** 1 Key Laboratory of Medical Virology, Ministry of Health; National Institute for Viral Disease Control and Prevention, China Centers for Disease Control and Prevention, Beijing 102206, China

**Keywords:** Human coronavirus, Spike protein, Indirect immunofluorescence assay, Antibody

## Abstract

**Background:**

Non-severe acute respiratory syndrome (non-SARS)-related human coronaviruses (HCoVs), including HCoV-229E, -HKU1, -NL63, and -OC43, have been detected in respiratory tract samples from children and adults. However, the natural prevalence of antibodies against these viruses in serum among population is unknown.

**Methods:**

To measure antibodies to the spike (S) protein of the four common non-SARS HCoVs, recombinant S proteins of the four HCoVs were expressed and characterised in 293 T cell. An S-protein-based indirect immunofluorescence assay (IFA) was then developed to detect anti-S IgG and IgM for the four individual HCoVs and applied to serum samples from a general asymptomatic population (218 children and 576 adults) in Beijing.

**Results:**

Of 794 blood samples tested, only 29 (3.65%) were negative for anti-S IgG. The seropositivity of the four anti-S IgG antibodies was >70% within the general population. The majority of seroconversions to four-HCoV positivity first occurred in children. Both S-IgG and S-IgM antibodies were detectable among children and increased with age, reaching a plateau at 6 years of age. However, no anti-S IgM was detected in healthy adults.

**Conclusion:**

Large proportions of children and adults in Beijing have evidence of anti-S IgG against four the HCoVs, and first infections by all four non-SARS HCoVs takes place during childhood.

## Background

Coronaviruses are positive-sense, single-stranded RNA viruses found in humans and a wide variety of animals [1]. In humans, four respiratory coronaviruses, namely human coronavirus (HCoV) -229E [2], -OC43 [3], -NL63 [4], and -HKU1 [5], are endemic worldwide. These four common strains cause diseases from mild, febrile upper respiratory tract illnesses to severe outcomes, such as croup, bronchiolitis, and pneumonia, and attribute to about 10% of all upper and lower respiratory tract infections in hospitalised children [1,6]. In 2003, a previously unknown coronavirus caused an outbreak of severe acute respiratory syndrome (SARS) in humans [7,8]. More recently, the novel betacoronavirus species HCoV-EMC was identified from a man with pneumonia in Saudi Arabia [9].

Coronaviruses are phenotypically and genotypically diverse [6,10]. All coronaviruses possess a common genome organisation in which the replicase gene encompassing the 5’ two-thirds of the genome is comprised of two overlapping open reading frames, ORF1a and ORF1b. The structural gene region, which covers the 3’ third of the genome, encodes the canonical set of structural protein genes in the order 5’-spike (S) → envelope (E) → membrane (M) → nucleocapsid (N)-3’ [1,10]. The S proteins is the most immunodominant CoV protein [1,6,10].

The most common method for diagnosing HCoV infection is reverse transcription polymerase chain reaction (RT-PCR) or real-time RT-PCR using RNA extracted from respiratory tract samples, such as nasopharyngeal aspirates[6,11,12]. On the other hand, serological assays for detection of antibody to HCoV are more complex to establish [1,6]. However, determining the levels of immunoglobulin M (IgM) and immunoglobulin G (IgG) antibodies in appropriate serum or plasma samples allows the researcher to define the point in time of primary infection as well as exposure rates; seroepidemiological studies are also important tools for HCoV infection diagnosis and research[6,13-17]. Previous reports have indicated that immunofluorescence assays (IFAs) for the detection of seroconversion with IgG antibodies against the main structural (N and S) proteins of SARS-CoV are useful for the diagnosis of acute SARS-CoV infection [18,19]. Little is currently known about the prevalence of anti-S antibodies specific for non-SARS HCoV infection among children and adults. We propose that an IFA for the detection of IgG against structural (N and S) proteins of non-SARS HCoV may be suitable for seroepidemiological studies.

To expand the epidemiological knowledge of four non-SARS-related endemic HCoVs in China, we expressed S proteins in a eukaryotic system and established an IFA for the detection of IgG or IgM antibodies against these four viruses. We used this system to determine the prevalence of anti-S IgG and IgM antibodies against HCoVs among a general population in Beijing.

## Methods

### Study population

794 serum samples were obtained from a general asymptomatic population (6 months to 75 years of age) who visited hospitals for medical examinations or vaccinations in Beijing from 1999 to 2011. All aspects of this study were performed in accordance with the national ethics regulations and approved by the Institutional Review Boards of the Centre for Disease Control and Prevention of China and the Ethics Committee of Peking Union Medical College Hospital. Participants received written information regarding the purpose of the study and of their right to confidentiality. Written informed consent was obtained from all participants or their guardians.

### Construction of HCoV-S expression plasmids

**Figure 1:**
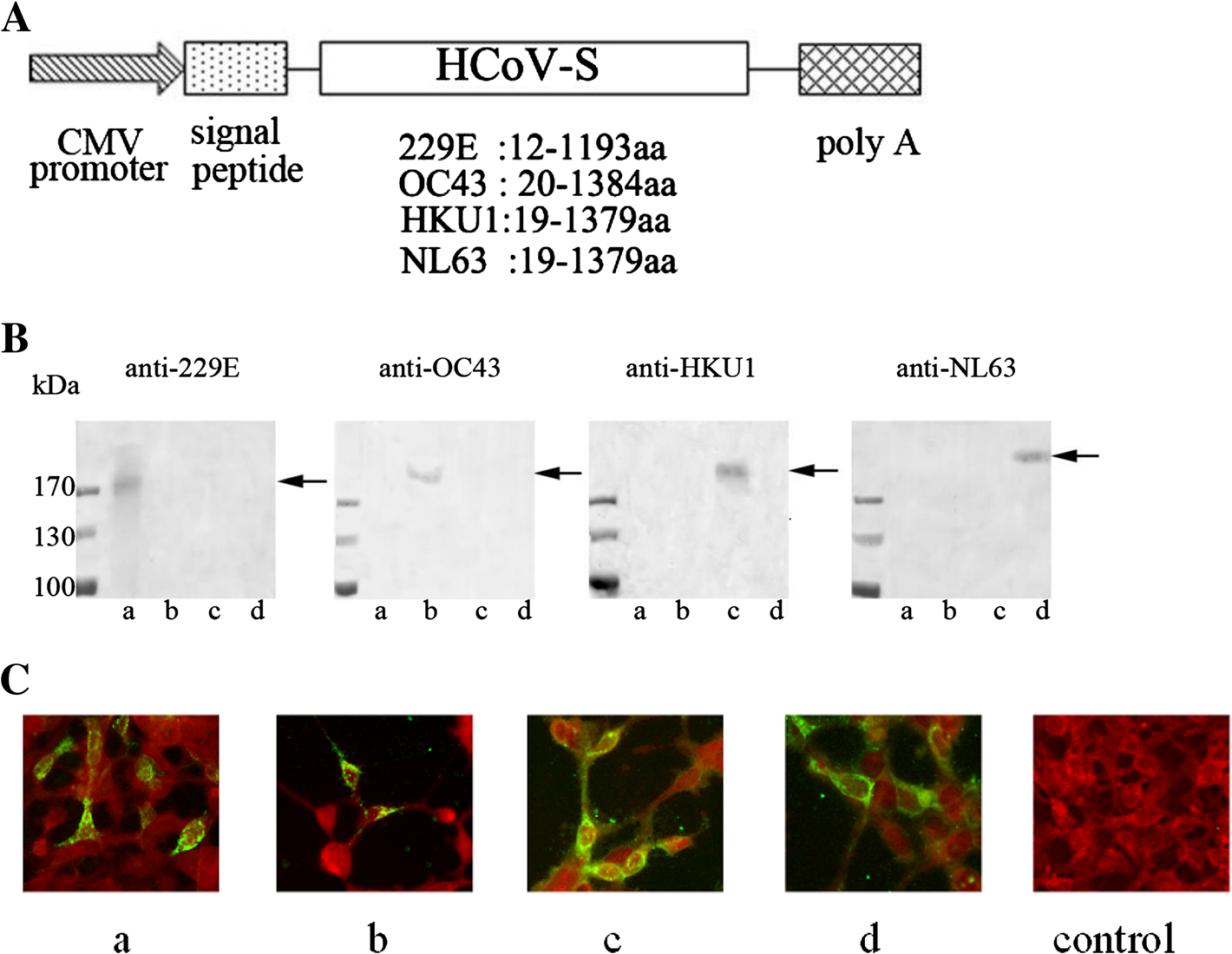
**Construction and characterization of spike expression plasmids derived from non-SARS HCoVs. (A)** Construction scheme of spike (S) fragment expression plasmids derived from four individual HCoVs (−229E, -OC43, -NL63, and -HKU1). **(B)** Characterisation of S proteins of individual HCoV expression plasmids by western blot using anti-S mouse serum from DNA vaccination. Forty-eight hours after 293 T cell transfection with the above individual HCoV expression plasmids, the cell lysates were collected for SDS-PAGE and western blot. **(C)** Characterisation of S protein by indirect immune fluorescence assay (IFA) using anti-S serum from mice immunised with individual HCoV expression plasmids. The control was transfected with pVRC-8303 plasmid and detected with mixed anti-S mouse serum of a non-SARS CoV.

S-protein-coding gene optimisation was conducted and oligonucleotides synthesised (Qingke Bio-Tech Engineering Service Co., Ltd., Beijing) according to the S-protein sequences of four non-SARS-related endemic HCoVs in GenBank (229ENC_002645, OC43NC_005147, HKU1NC_006577, and NL63NC_005831). The leader sequence of the original gene was removed and replaced by a human TPA leader sequence (1–21 aa) at the N-terminal of the S fragment. Artificially synthesised S fragments of HCoV-OC43, -229E, -HKU1, and -NL63 were cloned into the eukaryotic expression plasmid pVRC (a gift from Dr. Gary Nabel, NIH, USA) and named pVRC-229E-S, -OC43-S, -HKU1-S, and -NL63-S, respectively (Figure 1A). The plasmid pVRC-8304 (from Dr. Gary Nabel, NIH, USA) [20], in which the S-protein-coding gene of SARS-CoV was constructed and used as a DNA vaccine, was used as the control.

### Preparation of mouse anti-S sera by DNA immunisation

Female BALB/c (H-2d) mice between 6 and 8 weeks of age (Animal Care Centre, Chinese Academy of Medical Science, Beijing, China) were randomly assigned to four groups. In accordance with the Institutional Animal Care and Use Committee (IACUC)-approved protocol, all mice were immunised at weeks 0, 3, and 6 and bled at week 8. The mice were anaesthetised and primed with the above S-expression plasmid using intradermal injection into the lower dorsal side (10 μg/30 μL). Gene delivery using *in vivo* electroporation was performed as described previously [21]. Serum samples were collected 2 weeks after the last vaccination, and the pooled anti-S serum against individual HCoVs was stored at −70°C.

### Western blotting

293FT cells were transfected with individual S-expression plasmids using Lipofectinamine2000 reagent (Invitrogen Company). At 36 h post-transfection, the cells were lysed in ice-cold RIPA buffer (50 mM Tris–HCl [pH 7.5], 150 mM NaCl, 1% Triton X-100, 0.1% SDS, and 0.5% sodium deoxycholate) supplemented with a protease inhibitor mixture (Sigma, St. Louis, MO). The lysates were kept on ice for 10 min, centrifuged, and resolved by SDS/PAGE in a 6% polyacrylamide gel. The proteins were transferred to a nitrocellulose membrane, blocked with 5% skim milk in PBS for 2 h, and incubated for 8 h at 4°C with anti-S mouse polyclonal antibody diluted to 1:50 in blocking buffer. The membrane was washed in PBS containing Tween 20 (0.1%) and incubated for 1 h with horseradish peroxidase-conjugated anti-mouse secondary antibody (Pierce, Rockford, IL) diluted to 1:5000. The membrane was washed and the proteins were visualised with SuperSignal Chemiluminescence Substrate (Pierce).

### Indirect IFA

An IFA was used to detect HCoV S glycoprotein expression in 293 T cells. Briefly, 293 T cells seeded on glass slides were transfected with pVRC-229E-S, -OC43-S, -HKU1-S, and -NL63-S plasmids, respectively. After a 36-h incubation at 37°C in 5% CO_2_, the cells were fixed in 2% paraformaldehyde and blocked in 5% normal goat blood serum in 1% Triton-X-100 PBS. The infected cells were incubated with anti-S mouse serum (1:500) for 1 h, and then incubated with FITC-labelled goat anti-mouse IgG (H + L; Zhongahan Co., Beijing, China) for 30 min. Positive foci were identified by fluorescence microscopy (Nikon, Tokyo, Japan) after Evans blue duplicate staining.

For serum anti-S IgG or IgM detection using IFA, an individual HCoV S glycoprotein expression plasmid was used to transfect the 293 FT cells in the 75-cm^2^ flask. Forty-eight hours later, the transfected cells were washed twice with PBS and then dripped onto the slide. The cells were fixed using 4% paraformaldehyde for 10 min, then permeabilised using 0.2% TritonX-100 and washed three times with PBS. The anti-S-specific antibodies in sera (diluted to 1:20) were quantified using 1:100-diluted FITC-labelled sheep anti-human IgG (H + L; Zhongahan Co., Beijing, China) or 1:40-diluted FITC-labelled anti-human IgM (μ-chain-specific), and the slide was viewed under an inverted fluorescence microscope (Olympus, Tokyo, Japan). Serum samples that reacted with HCoV S protein at a dilution of >1:20 were considered positive for anti-S antibodies when duplicate or triple test was consistent. Furthermore, we confirmed that non-transfected 293 T cells or those transfected with the control plasmid (pVRC8304, which expresses the S protein of SARS-CoV) did not react with the human serum samples tested.

### Statistical analysis

Statistical analysis was performed using the Statistic Package for Social Science(SPSS) statistic 17 package with χ^2^-test and Fisher’s exact test. Differences between the mean values of each group were considered significant at p < 0.05 when assessed using Tukey’s test.

## Results

### Population characteristics

The demographic characteristics of the study subjects are summarised in Table 1. Of 794 patients subjects studied, 215 (27.08%) were infants or children <14 years of age; 457 (57.55%) were male. The average age of the adults and children was 28.75 ± 17.79 years. The majority of patients in the child population were 1 to 3 years of age (n = 134, 61.47%), and the majority of adults were 31 to 50 years of age (n = 363, 63.0%).

**Table 1:** Demographic characteristics and anti-S IgG detection of the study subjects

**Characteristic**		**Value (male/female)**	**%**
No. of subjects		794 (457/337)	
Mean age (y)		28.75 ± 17.79	
Age range (y)		0.5–75	
Group			
Child (<14 y)		215 (113/102)	
	<1 y	8 (5/3)	
	1–3 y	134 (68/66)	
	3–6 y	46 (28/18)	
	7–14 y	27 (12/15)	
Adult (≥14 y)		579 (344/235)	
	15–30 y	150 (76/74)	
	31–40 y	211 (132/79)	
	41–50 y	152 (91/61)	
	≥51 y	66 (45/21)	
Any IgG (+)	229E	665	83.75
	OC43	590	74.31
	HKU1	549	69.14
	NL63	580	73.05
All (−)		29	3.65

### Construction and characterisation of HCoV-S expression plasmids

The structures of the S-fragments of individual HCoV expression constructs used in this study are summarised in Figure 1A. Expression of the HCoV S proteins was confirmed by western blotting (Figure 1B). The segments were detected by anti-S mouse serum derived from DNA vaccination with individual HCoV S-expression plasmids. All four non-SARS-related S proteins showed band sizes that were consistent with the predicted molecular mass (150–200 kDa), presumably reflecting glycosylated forms of S protein. In addition, the expression of recombinant HCoV S proteins was further confirmed by immunofluorescence staining using specific anti-S mouse serum, as noted above (Figure 1C). All S proteins were expressed mainly in the cytoplasm and membrane. No cross-staining was observed when other HCoV antiserum was used as the primary antibody, specifically concerning virus-antibody pairs of subgroup 229E/NL63 (alphacoronaviruses) or OC43/HKU1(batacoronaviruses). Furthermore, no false positive/negative result was observed in our study (data not shown).

### Prevalence of anti-S IgG according to age group

**Figure 2:**
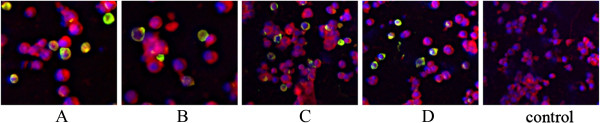
**Representative IFA for detection of anti-S antibodies of non-SARS HCoVs in human serum. A**, 229E-S expression plasmid; **B**, OC43-S expression plasmid; **C**, HKU1-S expression plasmid; **D**, NL63-S expression plasmid; control, mock plasmid pVRC-8304.

A representative anti-S-positive IFA result is shown in Figure 2. Using IFA based on 293FT cells expressing individual S proteins of HCoV, we investigated the seroprevalence of these viruses in serum from various age groups of a healthy population in Beijing. All samples were tested three using IFA and the result showed well reproducibility.

Of 794 serum samples tested for anti-S IgG by IFA, only 29 (3.65%) were negative for anti-S IgG of HCoV (Table 1). 665 (83.75%) serum samples were positive for HCoV-229E, 580 (73.05%) for HCoV-NL63, 590 (74.31%) for HCoV-OC43, and 549 (69.14%) for HCoV-HKU1. There were no significant differences between genders.

**Figure 3:**
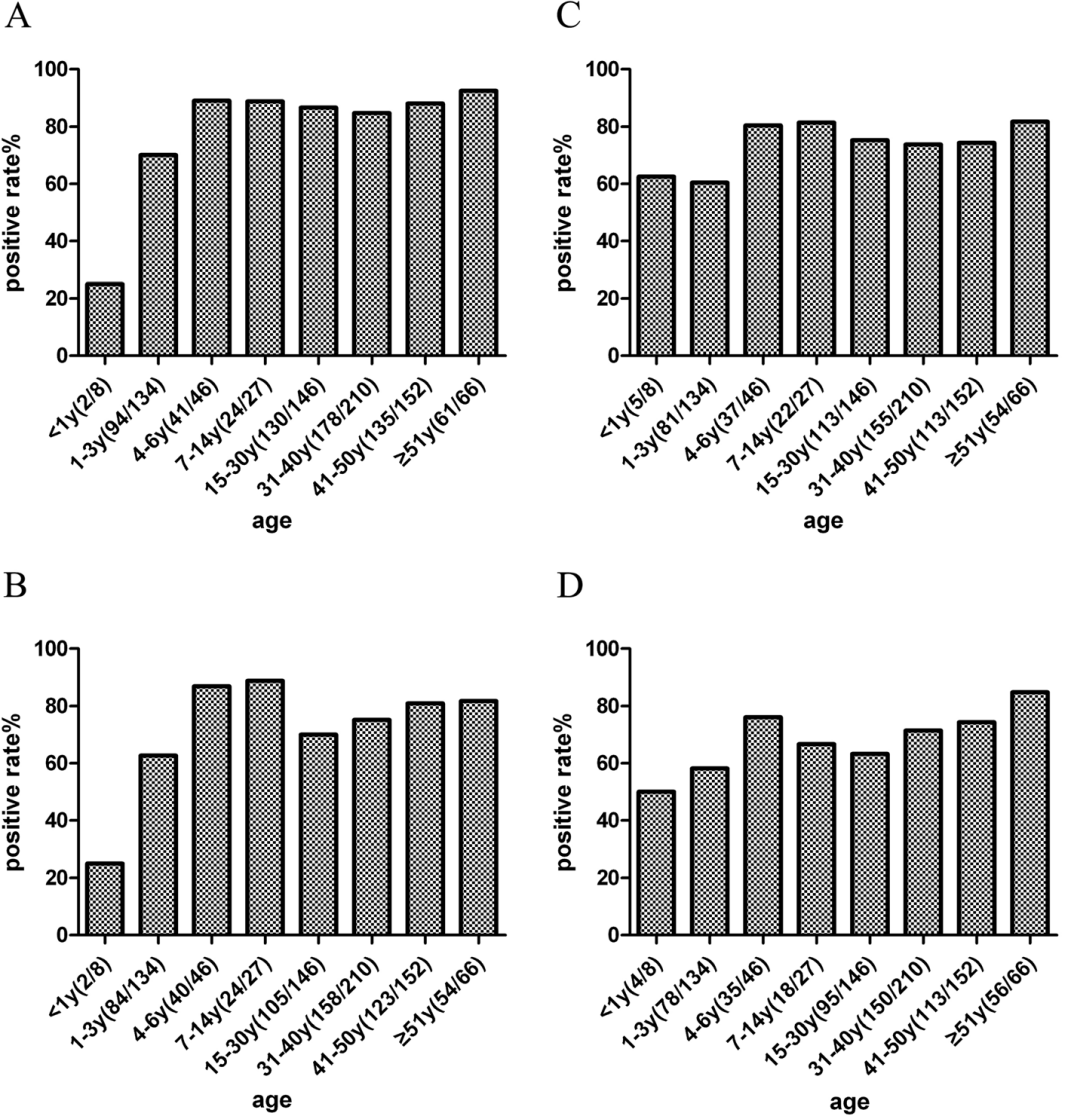
**Anti-S IgG positivity rate of individual HCoVs according to age group. (A)** Anti-S IgG positivity rates for HCoV-229E; **(B)** Anti-S IgG positivity rates for HCoV-OC43; **(C)** Anti-S IgG positivity rates for HCoV-HKU1; **(D)** Anti-S IgG positivity rates for HCoV-NL63.

The distribution and variation trend of anti-S IgG to the four HCoVs among the age groups are shown in Figure 3. The seropositivity rate was 70.15% for HCoV-229E and 62.69% for HCoV-OC43, which are similar to the levels of HCoV-HKU1 (58.21%) and HCoV-NL63 (60%) among the 1- to 3-year-old group (*p* > 0.05). The proportions of HCoV IgG-positive samples increased with age during infancy, reaching levels >75% at age >4 years. Interesting, a significant drop in seropositivity rate was observed for HCoV-OC43 in the 15- to 30-year-old group and for HCoV-HKU1 in the 7- to 14-year-old group. In the child population (n = 218), the total positivity rate of anti-S IgG antibodies was 73.85% for 229E, 68.81% for OC43, 61.93% for HKU1, and 66.51% for NL63 (Figure 3); no significant differences were observed with regard to IgG to two novel HCoVs among the children(NL63, *p* = 0.051; HKU1, *p* = 0.329).

In the adult population (≥14 years of age), the seroprevalence of anti-S IgG of HCoV-229E, -OC43, and -NL-63 did not differ significantly among the age groups (229E, *p* = 0.323; NL63, *p* = 0.545; OC43, *p* = 0.204). For HCoV-HKU1, a significant increase in the seroprevalence rate with increasing age was observed (*p* = 0.025). In the adult population (n = 576), the IgG positivity rate was 87.5% for 229E, 76.39% for OC43, 71.88% for HKU1, and 75.52% for NL63. The proportion of subjects with detectable IgG antibodies to HCoV-229E was significantly greater than that to -OC43 and -NL63 (*p* < 0.05). Furthermore, the number of subjects with IgG to the three above-mentioned HCoVs was also significantly greater than that to HCoV-HKU1 (*p* < 0.05). However, there were no significant differences in the anti-S IgG seropositivity rates of individual HCoVs with respect to gender in either the child or adult population (Data not shown).

### Anti-S IgM detection in children

**Figure 4:**
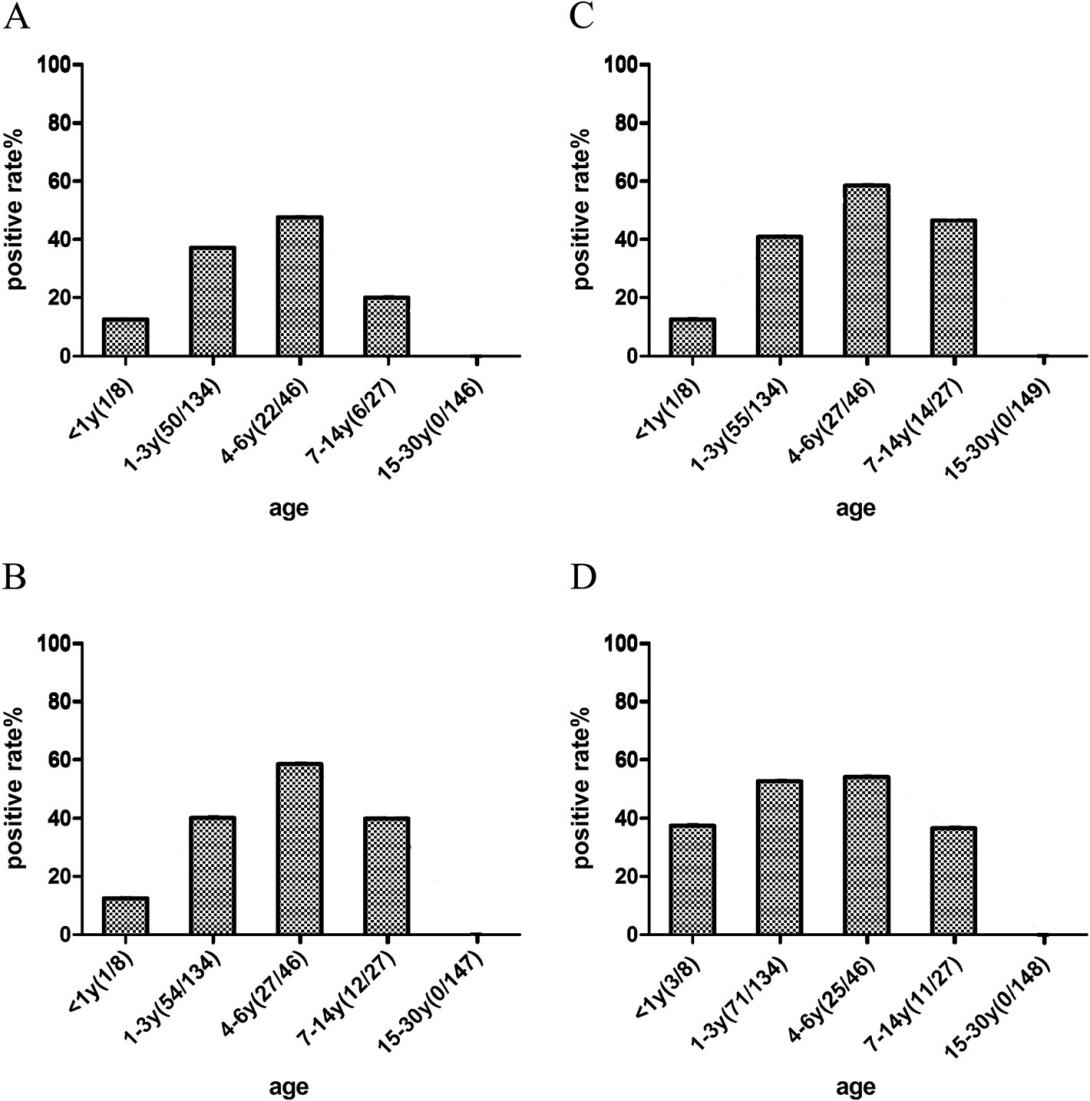
**Anti-S IgM positivity rates of individual HCoVs according to age group. (A)** Anti-S IgM positivity rates for HCoV-229E; **(B)** Anti-S IgM positivity rates for HCoV-OC43; **(C)** Anti-S IgM positivity rates for HCoV-NL63. **(D)** Anti-S IgM positivity rates for HCoV-HKU1.

The prevalence and variation trend of anti-S IgM antibodies to individual HCoVs among the child population is shown in Figure 4. The total positivity rate of anti-S IgM was 36.24% for 229E, 43.12% for OC43, 54.46% for HKU1, and 44.50% for NL63. The anti-S IgM positivity rate of HCoV-229E was significantly lower than that of the other three HCoVs (*p* = 0.028), while there were no significant differences in the anti-S IgM seropositivity rates of individual HCoVs with respect to gender (Data not shown).

There was a significant difference in the anti-S IgM positivity rate for individual HCoVs among age groups. The anti-S IgM positivity rate of HCoV-HKU1 was significantly higher than that of the other three HCoVs among the < 3-year-old groups (Figure 4). The positivity rate increased with age and began to drop after peaking in the school-age group (≥6 years), which is similar to that described above for anti-S IgG detection. No anti-S IgM antibodies for the four individual HCoVs were detected in the serum of the healthy adult population >15 years of age.

## Discussion

This is the first comprehensive study to evaluate anti-S IgG and IgM for four non-SARS HCoVs among the general population in China. We first time show that anti-S IgM is only present in children, indicating that for all viruses first infection takes place during childhood (age < 14 years).

The SARS epidemic that originated from southern China in 2003 sparked interest in all areas of coronavirus research [4-9]. Four endemic non-SARS-related HCoVs (HCoV-OC43,-229E,-NL63 and –HKU1) are major contributors to respiratory tract infections and other clinical manifestations [6,11-17,22]. However, specific and feasible serological surveys of these HCoVs, especially for HCoV-NL63 and –HKU1, have to date been reported among the general population only rarely in China. Spike is the major structural protein of HCoVs [1,10]. It contains multiple conformational epitopes that are major inducers of antibody neutralisation, and it has the lowest sequence conservation among coronavirus proteins, rendering it a specific target for serodiagnosis [1,10,23-26]. We chose the HCoV S gene for recombinant expression on the basis of current knowledge of immunodominant SARS-CoV antigens, which belongs to the same virus family as HCoV. Previous studies of SARS-CoV serology have successfully used the S protein in enzyme immunoassays, immunoblots, and IFA [23-27]. In addition, comparison of the S protein sequences of four HCoVs revealed that these proteins share <35% similarity [1,10]. We hypothesised that the difference in the amino acid sequence is sufficiently high to ensure the usefulness of S protein as a specific antigen for antibody detection. The native surface S protein expressed in 293 T cells can be recognised in post-infection population serum by IFA. Some reports indicated that this cell-based S protein expression system can differentiate false-positive ELISA results using the more cross-reactive nucleoprotein antigen[18,24]. Furthermore, Woo *et al*. reported that the rS-based IgM ELISA is more sensitive than the rN-based IgM ELISA for SARS-CoV pneumonia [25]. Our results showed that the S-based IFA enabled specific detection of IgG or IgM to four individual HCoVs.

Using IFA, we investigated the natural seroprevalence of four non-SARS-related HCoVs in blood samples from a general population that comprised a variety of age groups. Anti-S IgG antibodies to these four non-SARS-related HCoVs were detected at high rates (>70%) among healthy adults. Both anti-S IgG and IgM antibodies were found in the child group, and their prevalence increased with age up to 6 years, at which point it almost plateaued. These data suggest that exposure to HCoV is common in childhood and first infections by all four non-SARS HCoVs takes place during children. Moreover, we found evidence of anti-S IgG against double and multiple HCoVs in various combinations, which indicates that large proportions of the general population in Beijing may experience infections with more than one HCoV.

The seroprevalence of HCoV antibodies varies widely among studies, which used different antigens and methodologies [6,13-18,26-33]. Previous studies of non-SARS HCoVs demonstrated that seroprevalence varies greatly depending on the age of the population [6,13-18,26-33]. Our IFA study showed that the prevalence of the four anti-S IgG antibodies among this general Chinese population were slightly higher than those in another study from Germany using an *Escherichia coli* BL21-expressed recombinant N-based line immunoassay [13], which reported that the seropositivities in 25 healthy blood donors were 48% for HCoV-HKU1, 52% for -OC43, 56% for -229E, and 60% for -NL63. On the other hand, the seropositivity rate of three individual HCoVs excluding HCoV-HKU1 in this study was lower than that in another study of a US metropolitan adult population using baculovirus-expressed recombinant N-based ELISA [14], which showed seropositivity rates of 90.8% for HCoV-OC43, 91.3% for -229E, and 91.8% for -NL63, while that for -HKU1 was 59.2%. It was also supposed that the significantly different seropositivity rates for the various HCoVs might result in individuals with different demographic factors (*e*.*g*. ethnicity, smoking status, and socioeconomic status) having different susceptibilities to individual HCoVs [14]. A recent study of 105 older adult veterans with underlying chronic obstructive pulmonary disease at seven US sites showed that serum IgG to HCoV-229E, -NL63, and -OC43 was detected in at least 98% of subjects, while antibodies to -HKU1 were identified in 96 subjects (91%) [17]. Thus EIA assays that use whole virus or N protein as the antigen, using which apparent cross-reactivity of HCoV antibodies has been demonstrated previously [18,24], may detect group- rather than type-specific antibody.

Our results regarding anti-S IgG to four individual HCoVs in children correlate with those of previous seroprevalence studies [6,13-18,26-33], although the data on HCoV-NL63 and -HKU1 were limited. Shao *et al*. found that antibodies directed to HCoV-NL63 and -229E in children 1 year of age and older were frequently detected using part of the C-terminal region of the N protein as an antibody capture antigen in an ELISA [15]. A study of children in the Netherlands aged 2.5–3.5 years indicated that 75% and 65% of serum samples were positive for antibodies to HCoV-NL63 and -229E, respectively [16]. We observed that the prevalence of these four non-SARS-related HCoV-directed IgG antibodies among children >1 year of age were almost identical.

Information on the prevalence of anti-S IgM to non-SARS-related HCoV is to-date lacking. We first investigated the prevalence of anti-S IgM to four individual HCoVs among a general population in Beijing using IFA. Anti-S IgM to individual HCoVs was detected in a portion of the asymptomatic child population. The anti-S IgM seropositivity appeared to increase with age up to 6 years and decline sharply after 14 years of age. However, no anti-S IgM to individual HCoVs was detected in the healthy adult population. These results suggest that primary seroconversion to these viruses occurs mainly during childhood and youth; In addition, HCoV infection might result in seroconversion in children with asymptomatic or subclinical manifestations. These results are also in agreement with HCoV molecular epidemiological surveys [12,34], which have indicated that primary exposure occurs mainly in childhood and youth.

## Conclusion

We conclude that S-based IFA might be a useful specific serological platform for epidemiologic investigation of HCoV infection. High proportions of children and adults in Beijing show anti-S IgG seropositivity against the four HCoVs, and anti-S IgM antibodies were detected in the sera of asymptomatic children. These four non-SARS-related HCoVs appear be circulating in the general population, and sustained HCoV infection becomes more likely with increasing age. This study may serve as a basis for the prevention and control of non-SARS-related HCoV infection. However, further research is needed to determine the false-positivity and -negativity rates associated with this anti-S IFA by determining antibody titres during the acute and convalescent phases after primary HCoV infection. Comparison of serological methods and antigen preparations as well as sample exchange will facilitate validation of the assays for individual HCoV antibody determination.

## Abbreviations

(HCoVs): Human coronaviruses; (IFA): Indirect immunofluorescence assay; G (IgG): Immunoglobulin; M (IgM): Immunoglobulin; (RT-PCR): Reverse transcription polymerase chain reaction; (SARS): Severe acute respiratory syndrome; (S): Spike.

## Competing interests

The authors declare that they have no competing interests.

## Authors’ contributions

TW created the original idea of this research and designed the study. ZW and WW performed experiments. WH and LR provided important data analysis. ZW and TW drafted the manuscript. All authors read and approved the final version of the manuscript.

## Pre-publication history

The pre-publication history for this paper can be accessed here:

http://www.biomedcentral.com/1471-2334/13/433/prepub
